# Prevalence of Vitamin B12 Deficiency in Patients with Type 2 Diabetes Mellitus on Metformin: A Single Retrospective Study in a Tertiary Health Care Center Experience

**DOI:** 10.2174/0115733998400814250912060625

**Published:** 2025-10-08

**Authors:** Lina Bissar, Maha Khider Alghamdi, Sultan Alwajeeh, Lama Hefni

**Affiliations:** 1 Department of Internal Medicine, King Faisal Specialist Hospital & Research Centre, Jeddah, Saudi Arabia;; 2 Department of Psychiatry, Al Amal Hospital Jeddah, Jeddah, Saudi Arabia

**Keywords:** Post COVID-19 syndrome, Thai medicine, herbal steam bath, Metformin, antihyperglycemic medication, vitamin B12 deficiency

## Abstract

**Introduction:**

Globally, type 2 diabetes mellitus (T2DM) is a common metabolic disorder affecting millions of people annually. Metformin, a prevalent prescribed antihyperglycemic medication for T2DM, has been associated with vitamin B12 deficiency. This study aimed to determine the prevalence of vitamin B12 deficiency in T2DM patients receiving metformin.

**Methods:**

This retrospective record review study was based on T2DM patients receiving metformin at a single tertiary healthcare center. The prevalence of vitamin B12 deficiency is defined as a serum level <145 pmol/L. Statistical analysis assessed the association between vitamin B12 deficiency and metformin usage.

**Results:**

A total of 246 T2DM patients were included in the study. Among patients who used metformin, 7.7% were found to have vitamin B12 deficiency compared to 4.8% among those who did not use metformin, while the difference was not statistically significant (*p*=0.571). However, the median (IQR) level of vitamin B12 among metformin users (296 (186)) was significantly lower than that among patients who do not use metformin (382 (370)), *p*=0.001.

**Discussion:**

The study’s findings shed light on the important role of routine screening of vitamin B12 levels in patients on metformin, especially those on long-term use, for early identification and management of deficiency, leading to improved patient outcomes.

**Conclusion:**

Our study revealed a significantly lower level of vitamin B12 among metformin users in T2DM patients. However, the prevalence of vitamin B12 deficiency among metformin users did not differ significantly from those who do not use metformin.

## 1 INTRODUCTION

Type 2 diabetes mellitus (T2D) is one of the most common metabolic illnesses and is caused by two main factors: poor insulin secretion by pancreatic β-cells and inadequate insulin-sensitive tissue response to insulin [1]. The International Diabetes Federation (IDF) estimated that by 2021, approximately 537 million people worldwide will have diabetes mellitus [2]. A prior systematic review reported that the prevalence of T2D was 32.8% in Saudi Arabia, which is predicted to increase to 35.37% in 2020, 40.8% in 2025, and 45.8% in 2030 [3]. Globally, metformin, a drug from the biguanide class, is the most commonly used medication for T2D, with an estimated prescription of 120 million patients. It is effective, safe, and inexpensive [4]. Metformin has long been associated with vitamin B12 deficiency [5]. The Diabetes Prevention Program Outcomes Study (DPPOS) suggested that periodic testing of vitamin B12 levels should be considered in metformin-treated patients, especially those with anemia or peripheral neuropathy [6]. Almatrafi *et al*. conducted a study in 2022 and reported that 17.5% of patients with T2D on metformin had vitamin B12 deficiency [7]. Ting *et al*. also focused more attention on the dose and duration of metformin use, demonstrating a direct correlation with a decreased serum level of vitamin B12 [8]. These results are similar to those reported by Marar *et al.*, who reported a high prevalence of vitamin B12 deficiency in patients with T2D on metformin and suggested that the measurement of vitamin B12 should be a mandatory component of every patient's annual evaluation of T2D on metformin therapy [9]. Researchers have reported a correlation between long-term metformin use and decreased vitamin B12 levels. However, a significant amount of variation in metformin-induced vitamin B12 deficiency has been reported in the literature; thus, the present study aimed to evaluate the prevalence of vitamin B12 deficiency in patients with T2D who are using metformin compared with patients with T2D who are not using metformin.

## 2 METHODS

### 2.1 Study Setting and Participants

This retrospective chart review included all adult patients (≥18 years) with type II diabetes at King Faisal Specialist Hospital and Research Centre (KFSH&RC) in internal medicine clinics over 12 months to measure the prevalence of vitamin B12 deficiency in two groups: patients with type II diabetes on metformin vs. patients with type II diabetes and not on metformin. We excluded patients who had the following conditions: type 1 diabetes, initiation of insulin within 1 year of diagnosis, prior vitamin B12 injections within the past year, prior diagnosis of pernicious anemia (based on intrinsic factors), celiac disease, inflammatory bowel disease, a gastric balloon or (adjustable) band, tube placement for weight loss (Aspire, Assist, *etc.*), or gastrointestinal surgery (including metabolic surgery, gastrectomy, and colectomy). Consent forms were obtained from the patients before starting the study, and the collected data were kept confidential. The cutoff levels for vitamin B12 are as follows:

A vitamin B12 concentration < 145 pmol/L is deficient, a vitamin B12 concentration ranging from 145-200 pmol/L is low or borderline, and a vitamin B12 concentration >500 pmol/L is considered abnormally high.

The following data were extracted from the medical records of the hospital's electronic system (Powerchart in Citrix software): medical record number, age, sex, nationality, body mass index (BMI), duration of DM, duration of use of metformin, dose of metformin in mg (<1000, 1000-1500, >1500), date of metformin discontinuation if any, presence of peripheral neuropathy (tingling or numbness sensation in hands or feet), use of any of the following (B12 supplements or B complex, multivitamins, H2 blockers, proton pump inhibitors (PPI), antacids), presence of any diet restriction (diabetic diet, low-fat diet, vegan, vegetarian, meat restriction, other), hemoglobin level, mean corpuscular volume, vitamin B12 level, or intrinsic level (positive, negative, unknown).

### 2.2 Statistical Analysis

We performed our analysis *via* STATA 18, from which descriptive statistics and frequency tables were derived. The quantitative continuous variables were compared *via* the Mann‒Whitney U test and Pearson correlation coefficient.

Fisher’s exact test was used to compare the prevalence of vitamin B12 deficiency between the two groups. We also performed a simple linear regression test to compare two continuous variables.

## 3 RESULTS

We used STATA 18 to analyze our data, and we determined a *p*-value of 0.05 to be a point to determine whether the null hypothesis was accepted or rejected. After we excluded patients who met the exclusion criteria, 246 patients were included. The patients were almost equal in sex distribution (50.81% males and 49.19% females). The mean age of the patients was 63 years; the youngest patient was 22 years old, and the oldest patient was 90 years old.

The mean vitamin-B12 level was 369, with a minimum of 25 and a maximum of 1444. The participants had a mean BMI of 32, and approximately 73% of them were hypertensive. In addition, 74.18% of the participants were taking metformin, and 25.82% of them were not. The mean duration of metformin use is 13 years, with a minimum duration of 1 year and a maximum duration of 30 years. Approximately 8% of those who took metformin were taking a dose of less than 1,000 mg, 29% were between 1,000-1,500 mg, and 61% were taking more than 15,000 mg (Fig. **1**). Additionally, 7% of the patients were vitamin B12 deficient, 13% had a low (borderline) level, 63% had a normal level, and approximately 15% had vitamin B12 levels higher than the cutoff level at our hospital laboratory. Among patients who used metformin, 7.7% were found to have vitamin B12 deficiency, which did not differ significantly (*p*=0.571) from those who did not use metformin (4.8%). 2% of the participants were positive for antibodies against intrinsic factors, and 17% of the participants suffered from peripheral neuropathy.

Patients on metformin had significantly lower median serum vitamin B_12_ levels (296 pg/mL) compared to non-users (382 pg/mL, *p* = 0.001). We performed the Shapiro‒Wilk test to examine the normality of the dependent variable (B12 level), and the *p* value was less than 0.001, which indicates that the variable was not normally distributed (Fig. **2**). Given that the dependent variable (vitamin B12) is not normally distributed, we used nonparametric tests to compare the mean vitamin B12 between those who take metformin and those who do not. The mean B12 level among those who take metformin is 332, whereas the mean B12 level among those who do not is 478. The Wilcoxon rank-sum (Mann‒Whitney) test resulted in a *p*-value of 0.0005, which indicates that the difference in the mean is statistically significant. Another Wilcoxon rank-sum (Mann‒Whitney) test was performed to compare the vitamin B12 level between males and females, but the *p*-value was 0.55, which revealed that there was no statistically significant difference in the vitamin B12 level between males and females (Fig. **3**). The Pearson correlation coefficient between the age of the participants and the level of B12 is 0.03, which indicates a weak positive relationship, but it is not statistically significant (*p*-value of 0.62).

Approximately 17% of our patients state that they have peripheral neuropathy when they are asked about their experience of tingling, numbness, or pins in their periphery. The mean vitamin B12 level among those who do not complain of peripheral neuropathy is 360, whereas the mean vitamin B12 level among those who have peripheral neuropathy is 411. In addition, 7.8% of those who did not have peripheral neuropathy were found to have vitamin B12 deficiency, whereas only 4.7% of those who were confirmed to have peripheral neuropathy were found to be vitamin B12 deficient.

To examine the relationship between the duration of metformin use and the level of vitamin B12, we performed a simple linear regression test between the duration of metformin in months and the level of vitamin B12. The coefficient is 0.224, and the *p*-value is 0.103, which is not statistically significant. We also performed a Pearson correlation test between the duration in months and the level of B12; the Pearson correlation coefficient was 0.117, and the *p*-value was 0.102, which was again statistically not significant.

## 4 DISCUSSION

Vitamin B12 deficiency has serious consequences, such as peripheral neuropathy, myelopathy, and megaloblastic anemia [10]. Furthermore, one may confuse peripheral neuropathy resulting from vitamin B12 deficiency with diabetic neuropathy [11]. Our results are in accordance with those of previous studies that reported a decrease in vitamin B12 with the use of metformin. We found a statistically significant (*p* value < 0.001) difference between the mean B12 level of those who took metformin and those who did not, which is consistent with the findings of numerous previous studies, including those by Ting *et al.*, Hermann *et al.*, and Pflipsen *et al*. [8, 12, 14]. This difference was 243 pmol/L between the two groups, which is clinically significant. We found that 7.7% of the T2D patients who were taking metformin were vitamin B12 deficient, which is also consistent with what Pflipsen *et al.* reported; they reported that 7% of their patients who were taking metformin were vitamin B12 deficient. However, we subdivided our patients into two groups: deficient and low (borderline). We found that 23% of our patients had decreased B12 levels, both deficient and low, whereas 11% of our participants did not take metformin. Both numbers—the deficient and the borderline low—are significant and consistent with what others found. The percentages also might change from one study to another according to the patient population and the cutoff point for vitamin B12 deficiency. We used values below 145 for deficient and below 200 for low.

We performed a Pearson correlation between the vitamin B12 level and the duration of metformin in months, and we found a weak positive correlation (Pearson correlation 0.112), which was statistically insignificant. This positive correlation contrasts with what Marar *et al*. reported, as they both reported a statistically significant relationship between the duration of metformin use and vitamin B12 deficiency [9]. The negative relationship makes sense, as increased exposure to metformin leads to a decrease in vitamin B12 absorption. Our study did not find this relationship, probably because of the smaller sample size, as the previous two studies had a larger sample size to generate enough power to detect the difference.

Peripheral neuropathy was one of the variables we examined in our patients, and this has also been investigated in previous studies by Alvarez *et al.*, Gupta *et al.*, Gosh *et al.*, and Alharbi *et al*. [13, 15-17], which revealed a statistically significant inverse relationship between vitamin B12 levels and peripheral neuropathy. Interestingly, patients with peripheral neuropathy in our study had higher serum vitamin B12 levels compared with those without neuropathy. This finding contrasts with previous reports linking metformin use to lower B12 levels and an increased risk of neuropathy. Patients with peripheral neuropathy may have poorer glycemic control, which itself might lead to diabetic neuropathy regardless of the vitamin B12 level. Diabetic peripheral neuropathy is one of the most common chronic complications of diabetes, affects patients’ quality of life, and has a significant cost to the health care system [18, 19].

Previous studies also revealed significant improvement in peripheral neuropathy after screening and supplementation with vitamin B12 [20, 21]. We included risk factors such as age, duration, and the dose of metformin; however, we could not find a statistically significant association, even though previous studies reported some correlations with risk factors such as age, dose, and duration [19, 22].

It is important to acknowledge the limitations of this study, as it is a single-center retrospective study. Therefore, this study is subject to the limitations inherent to retrospective studies, including missing data in some patients’ files.

## LIMITATIONS

Although our study has reached its main aim, some limitations of this study should be highlighted. It is a single-center, retrospective study; thus, it has all the limitations inherent to retrospective studies, including incomplete data or limited documentation in some patients, which makes it hard to obtain relevant information.

## CONCLUSION

Our results indicate that our patient population needs a periodic vitamin B12 checkup, as those who are taking metformin showed both statistically and clinically significant decreases in vitamin B12 levels. We recommend regular vitamin B12 level checkups in metformin-treated diabetic patients to allow for early detection and timely management of deficiency.

## Figures and Tables

**Fig. (1):**
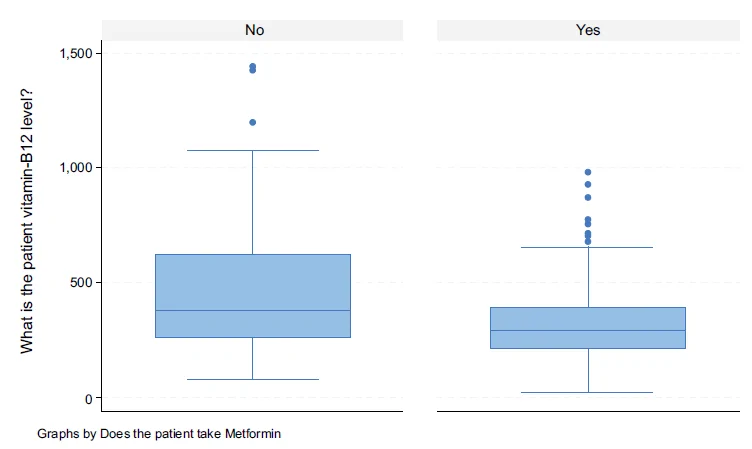
Comparison of serum vitamin B12 levels between patients taking Metformin and those not taking Metformin.

**Fig. (2):**
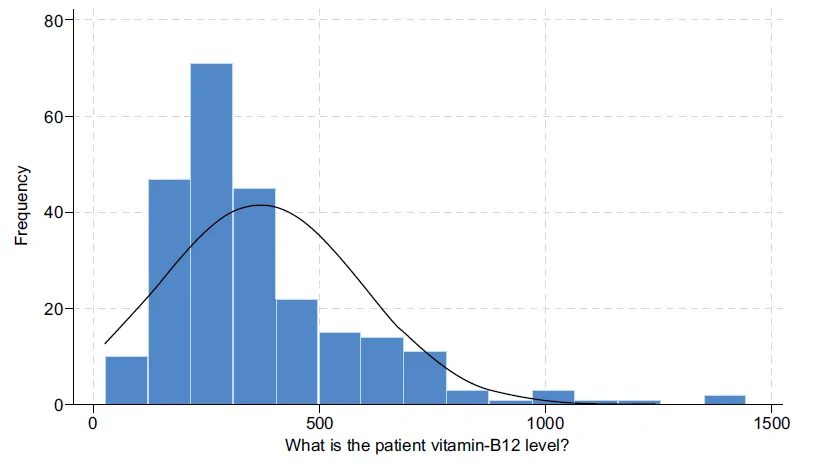
The distribution of serum vitamin B12 levels among patients.

**Fig. (3):**
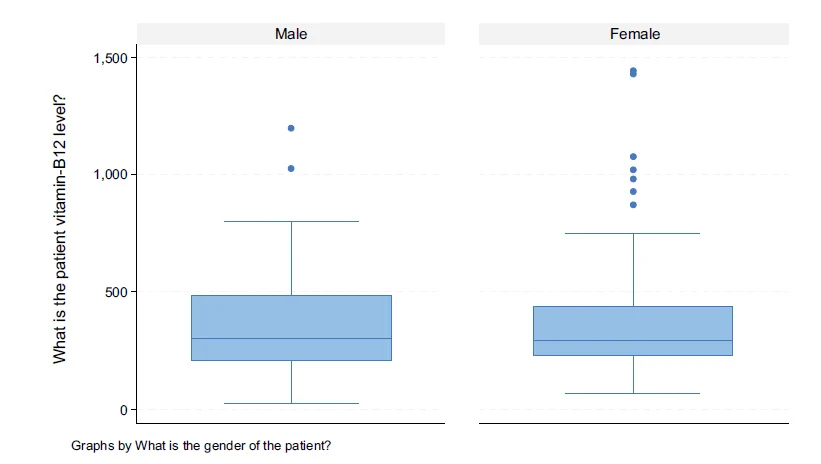
Comparison of serum vitamin B12 levels between male and female patients.

## Data Availability

The raw data will be provided in an Excel sheet upon reasonable request.

## LIST OF ABBREVIATIONS

T2DM: Type 2 Diabetes Mellitus
DPPOS: Prevention Program Outcomes Study
BMI: Body Mass Index
KFSH&RC: King Faisal Specialist Hospital and Research Centre

## STANDARDS OF REPORTING

STROBE guidelines were followed.
